# Der Tanz der kleinen Spirochäten: Theatrale Hygieneaufklärung und die Bekämpfung der Geschlechtskrankheiten in der frühen Sowjetunion

**DOI:** 10.1007/s00120-020-01133-9

**Published:** 2020-02-11

**Authors:** O. Kosenko, I. J. Polianski

**Affiliations:** Institut für Geschichte, Theorie und Ethik der Medizin, Universität Ulm, Parkstraße 11, 89073 Ulm, Deutschland

**Keywords:** Medizingeschichte, Theater, Gesundheitliche Aufklärung, Syphilis, Sexuell übertragbare Infektionen, History of medicine, Theatre, Health education, Syphilis, Sexually transmitted infections

## Abstract

Der vorliegende Beitrag untersucht antivenerische Theateraufführungen als Mittel zur Bekämpfung von Geschlechtskrankheiten in der frühen Sowjetunion. Er fragt danach, in welchen Bildern, Figuren und Handlungen das venerologische Wissen auf der Theaterbühne zur Darstellung kam, welche Genretraditionen und kommunikative Mittel benutzt wurden sowie wie das Publikum und die Theaterkritik das Bühnengeschehen aufnahm. Zu diesem Zweck werden Archivquellen, ausgewählte Texte antivenerischer Gerichtsspiele und Dramen sowie Berichte und Rezensionen in der Tagespresse ausgewertet.

## Abstract (en)

The present contribution analyses the antivenereal theatre performances as a means of fighting sexually transmitted diseases in the former Soviet Union. It is asked which images, figures and actions regarding venereal knowledge were presented on stage, which genre traditions and communicative instruments were used, and how the audience and theatre critics perceived the events on stage. For this, the archive sources, selected texts of antivenereal mock trial plays and dramas, reports and reviews in the daily press have been evaluated.

## Einleitung

In der Zeit um die Wende des 20. Jahrhunderts hat sich der gesellschaftliche Umgang mit Geschlechtskrankheiten grundsätzlich verändert. Sie wurden zunehmend enttabuisiert und rückten in den Fokus einer breiten Öffentlichkeit [1]. Was bisher ein Privatproblem des Einzelnen war, verwandelte sich in eine zeitdiagnostische Metapher, soziale Herausforderung und Bedrohung. In der Literatur des „fin de siècle“ finden sich vermehrt Werke, die um das Thema Geschlechtskrankheiten kreisen. Sie avancierten hier zu einem angstbesetzten Attribut des Molochs Großstadt, geprägt durch ausschweifende Lebensweise, sinnlichen Genuss und käufliche Liebe [2]. Mit *Les Avariés* (Die Schiffsbrüchigen) Eugène Brieuxs (1858–1932) von 1901, dem meist diskutierten Theaterstück in diesem Jahrzehnt, betrat das Thema die theatrale Bühne [3]. Die zeitgleich aufkommende antivenerische Bewegung griff solche dramaturgischen Vorlagen auf und setzte sie für ihre sozialhygienischen Aufklärungs- und Abschreckungskampanien gezielt ein. So wurde das Drama von Brieux in Deutschland auf Initiative der Deutschen Gesellschaft zur Bekämpfung der Geschlechtskrankheiten als Aufklärungsstück auf die Theaterbühne gebracht [4]. Der Einsatz theatraler Inszenierungen zu Zwecken antivenerischer Aufklärung hat in den westeuropäischen Ländern nie einen systematischen Charakter angenommen. Allerdings diente ihre Erfolgsgeschichte als Vorbild für die theatrale Hygienepropaganda in der jungen Sowjetunion, wo sie eine fest institutionalisierte Form erhielt. In den 1920er-Jahren wurden in Moskau und anderen größeren Städten des Landes spezielle Theater der sanitären Aufklärung eröffnet. Blickt man auf die thematischen Schwerpunkte dieser damals sog. „sanitären Rampe“, so fällt sofort auf, dass der Bekämpfung der Geschlechtskrankheiten ihre meiste Aufmerksamkeit galt. Diese antivenerischen Inszenierungen sind der Untersuchungsgegenstand des vorliegenden Beitrags.

Abgesehen von einigen Arbeiten, die sich unter theaterwissenschaftlichen [5–9] oder rechtsgeschichtlichen [10, 11] Fragestellungen mit den sog. Agitationsgerichten befassen, wurde die antivenerische Aufklärung auf der Bühne medizinhistorisch bislang nicht erforscht. Von dieser Forschungslücke ausgehend untersucht der Beitrag, in welchen Bildern, Figuren und Handlungen das venerologische Wissen zur Darstellung kam. Welche Genretraditionen und kommunikativen Mittel kamen zum Einsatz? Wie wurde das Bühnengeschehen im Publikum aufgenommen, in der Tagespresse kommentiert und von der Theaterkritik bewertet? Zur Beantwortung dieser Fragen stützt sich der Beitrag im Wesentlichen auf archivalische Überlieferungen. Darüber hinaus wird auf gedruckte Quellen zurückgegriffen: publizierte Theaterstücke, Presseberichte und Rezensionen.

## Die sanitäre Rampe

Das politische „Heilsziel“ eines „neuen Menschen“, das sich die bolschewistische Disziplinarmacht auf die Fahnen schrieb, erforderte sowohl geistige als auch körperliche Erneuerung und damit eine hygienische Optimierung der Massen. Zu diesem Zweck wurden seit Beginn der 1920er-Jahre auf Freilichtbühnen und in den Klubhäusern, in Fabrikhallen und Lesehütten für Arbeiter und Bauern und selbst auf den Kolchosfeldern der Sowjetrepublik Agitprop-Revuen, inszenierte Agitationsgerichte, „Lebendige Zeitungen“ und Lehrstücke aufgeführt, die aktuelle sozialmedizinische Probleme thematisierten. Allgegenwärtig auf der „sanitären Rampe“ war die Figur des Arztes, der beispielsweise als Conférencier im weißen Kittel in den Pausen auftrat, um das Bühnengeschehen einzuleiten oder fachgerecht zu kommentieren [12].

Ein Großteil solcher Darbietungen wurde von den Organen des Volkskommissariats für Gesundheitsschutz initiiert und veranstaltet. 1921 wurde in diesem institutionellen Rahmen in Moskau unter der Leitung von Theaterregisseurin Ol’ga V. Rachmanova (1871–1943) ein Künstlerisch-Dramatisches Theaterstudio ins Leben gerufen [13]. Die Aufführungen dieses Theaterstudios stießen auf so große Resonanz, dass selbst der namhafte Theaterreformer und Regisseur Konstantin S. Stanislavskij (1863–1938) über die engagierte Arbeit dieses Theaterstudios sich in einem Gutachten begeistert äußerte [14]. Auf der Welle dieses Erfolgs entstand schließlich in Moskau ein eigenes Hygienetheater. Das Experimentelle Theater der Sanitären Aufklärung (später Staatliches Wandertheater der Sanitären Kultur, TSK) nahm am 27. Januar 1925 erstmals offiziell den Spielbetrieb auf und bereits am 5‑jährigen Jubiläum seit der Eröffnung konnte sich sein Direktor damit rühmen, dass sich sein Theatervorhang zum 1200. Male geöffnet habe. Laut anderen Angaben, die auch Gastspielreisen des Theaters berücksichtigen, hat es allein von 1925 bis 1928 1300 Aufführungen gegeben, die mehr als 1 Mio. Zuschauer besuchte [15]. Das Beispiel machte Schule in Leningrad, Perm, Tiflis, Lugansk, Odessa und gab einen mächtigen Impuls für die Schaffung von zahlreichen sanitären Amateurtruppen, die diese Form der kollektiven „Theatertherapie“ dem Publikum ebenfalls anboten. Allein in der Ukraine eröffneten bis 1932 sechs sanitäre Theater ihre Tore [16].

Das „sanitäre Repertoire“ reichte von sog. sanitären Agitgerichten, in denen Kurpfuscher, Trinker, Prostituierte und selbst Mücken und Bakterien sich vor Gericht verantworten mussten, über revueartige „lebendige Zeitungen“, die mithilfe von Agitpropdichtung und akrobatischen Einlagen vom Geschehen an der „sanitären Front“ berichteten, bis hin zu Ein- und Dreiaktern zu medizinischen Themen [17]. Dabei arbeiteten selbst namhafte Autoren wie Michail A. Bulgakov im Auftrag des TSK. Thematisch galt die absolute Priorität „sozialen Pathologien“ und allen voran den Geschlechtskrankheiten.

## Sexualität und Geschlechtskrankheiten in der frühen Sowjetunion

Die intensive Beschäftigung mit Geschlechtskrankheiten ist im Kontext einer Sexualrevolution zu sehen, die sich nach der Oktoberrevolution von 1917 im Sowjetrussland gegen die „rückständige Sexualmoral“ und „scheinheilige Prüderie“ herrschender Klassen richtete und auf eine Abkoppelung von Sexualität und Fortpflanzung abzielte [18]. Stattdessen wurden die Ideale der freien Liebe, solidarischen Lebensgemeinschaft in der Kommune und kollektiven Kindererziehung propagiert [19].^1^ Seit 1920 sah sich Sowjetrussland gerne im Lichte seiner Errungenschaft, als erster Staat der Welt den Schwangerschaftsabbruch auf Wunsch der Frau legalisiert zu haben.^2^ Strenge Reglementierung und strafrechtliche Verfolgung der Prostitution wurde außer Kraft gesetzt. Während der Phase des Kriegskommunismus (1918–1921) wurden die Prostituierten allerdings als „soziale Parasiten“ und „Deserteure der Arbeitsfront“ massenhaft verhaftet und interniert [24]. Mit dem Ende des Bürgerkriegs und der Kehrtwende vom Kriegskommunismus zur relativen Zurückhaltung politischer Gewalt und partiellen Tolerierung marktwirtschaftlicher Verhältnisse (die sog. neue ökonomische Politik, NÖP) ab 1921 hörten diese drakonischen Maßnahmen jedoch auf. Die gewerbliche Unzucht durfte zwar aus klassenpolitischen Gründen nicht toleriert werden, weil sie als in privatkapitalistischen Machtverhältnissen verwurzeltes soziales Übel, ein „Schatten des Kapitalismus“, anzusehen war [25]. Die Prostituierten selbst wurden von nun an aber als unschuldige Opfer und Überbleibsel des zaristischen Regimes eher bemitleidet als kriminalisiert [24].

**Figure 1:**
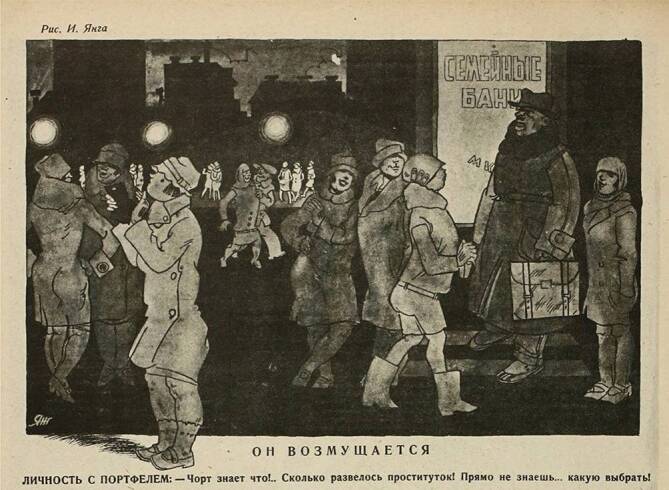

Die NÖP bot insbesondere in den Städten neue Möglichkeiten und Räume der Unterhaltung und Freizeitgestaltung: Es eröffneten wieder Bars, Lichtspieltheater und Tanzlokale, wo vermehrt Frauen ohne geregeltes Erwerbseinkommen verkehrten. In St. Petersburg hat sich innerhalb des ersten NÖP-Jahres die Zahl der Prostituierten fast verdoppelt (Abb. 1 und 2; [26]). Bald kam in diesem geistigen Umfeld die berüchtigte „Glaswassertheorie“ auf, wonach den Geschlechtstrieb zu befriedigen eine ähnlich triviale Angelegenheit darstelle, wie ein Glas Wasser zu trinken. Dies sorgte in den Reihen der Sozialhygieniker für große Unruhe, die befürchteten, dass die ausufernde bolschewistische Freizügigkeit im sexuellen Bereich zu einer Massenverbreitung von sexuell übertragbaren Krankheiten führen würde.

**Figure 2:**
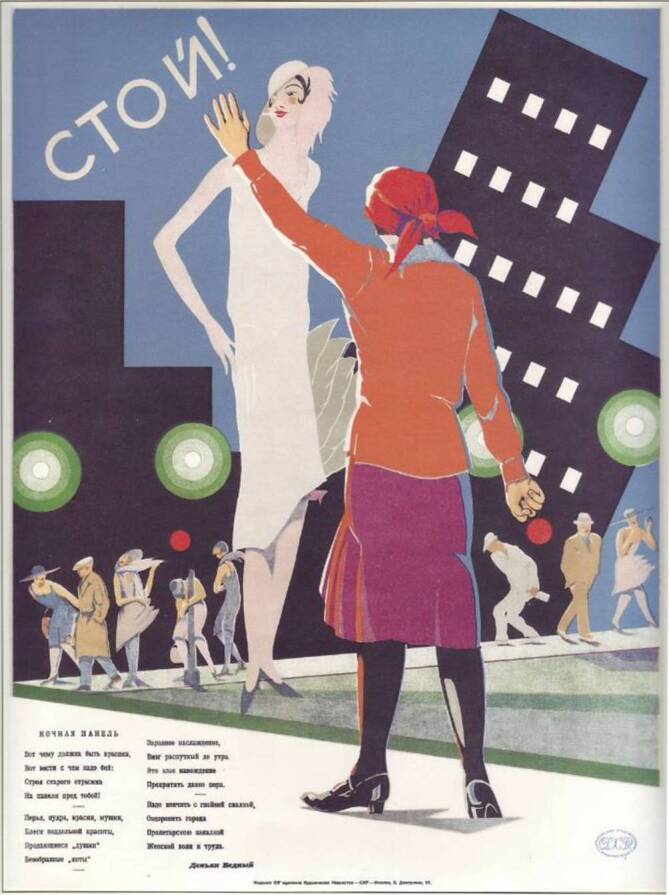

Ein Blick in die entsprechenden Statistiken zeigt allerdings, dass die große Katastrophe ausblieb. So bewegten sich im Russischen Imperium die Fallzahlen für Lues im Jahr 1913 nach Angaben der meisten Autoren zwischen 70–80 registrierten Fällen auf 10.000 Einwohner, wobei die Stadtbewohner mit durchschnittlich 180 Lueskranken je 10.000 um das 3‑Fache schwerer betroffen waren als die Landbevölkerung [27, 28]. Mit 81 registrierten Syphiliskranken auf 10.000 Einwohner im Jahr 1922 haben sich die Verhältnisse kaum verändert [28]. Die Infragestellung der bürgerlichen Sexualmoral hat allerdings im veränderten Ansteckungsmodus ihre Spuren hinterlassen. Hatte sich 1914 in Moskau mit 56,9 % noch der Großteil der männlichen Luespatienten bei Prostituierten infiziert und 39,6 % bei Gelegenheitsbekanntschaften, Bekannten oder Ehefrauen, bietet sich in den Jahren 1922–1924 ein verkehrtes Bild: 61,5 % steckten sich im Bekanntenkreis an, während nur noch 31,7 % der Ansteckungen auf die käufliche Liebe entfiel [27, S. 18]. Das Ansteckungsrisiko hat sich über alle Gesellschaftsschichten lediglich gleichmäßiger Verteilt, ohne im Wesentlichen abgenommen zu haben. Etwa ab Mitte der 1920er-Jahre zeichnet sich allerdings eine generelle Abnahme der Geschlechtskrankheiten ab. Die Erkrankungsrate der Syphilis sinkt 1926 allgemein auf 58 und in den Städten auf 120 auf je 10.000 Einwohner [28]. In St. Petersburg etwa, die 1913 mit 290 Luesfällen auf 10.000 zu den am meisten durchseuchten Großstädten zählte, findet man mit 227 für 1924, 107 für 1926 und 73 für 1927 rückläufige Fallzahlen [27]. Ähnliche Dynamik wurde bei Tripper beobachtet [26, S. 117]. Trotzdem blieb die Erkrankungsrate der Geschlechtskrankheiten bis in die 1930er-Jahre hinein auf sehr hohem Niveau, was sich wiederum in den Anstrengungen der Hygieneaufklärer spiegelt, ihre Ausbreitung einzudämmen.

## Die antivenerischen Gerichtsspiele

Die ersten Gerichtsinszenierungen gehen auf die Zeit des russischen Bürgerkriegs zurück. Als Vorbild dienten die in der Roten Armee abgehaltenen echten Militärtribunale gegen Deserteure, deren Zahl im Frühling 1920 explodierte. Da es dabei weniger auf die Bestrafung von einzelnen Fahnenflüchtigen ankam als vielmehr auf die Festigung der Kampfmoral der Rotarmisten, wurde bald ein fiktiver Deserteur und damit das Phänomen der Desertion als solches vor ein inszeniertes Gericht gezerrt. Es folgten weitere Agitgerichte, in denen politische, sanitäre oder landwirtschaftliche Delikte verhandelt wurden [11, S. 12, 50-52,70, 71].

Die sowjetischen Protagonisten der Hygieneaufklärung maßen sanitären Agitgerichten große Bedeutung bei. Anders als die herkömmlichen, meistens trocken wirkenden sanitären Vorträge fesselten sanitäre Gerichtsspiele die Aufmerksamkeit des Publikums. Manche von ihnen zogen Tausende begeisterte Zuschauer an und dauerten mehrere Stunden. Ihre verhaltensändernde Wirkung zeigte sich unmittelbar, indem sich direkt nach der „Urteilsverkündung“ vor den venerologischen Dispensaires Schlangen aus besorgten Arbeitern und Bauern bildeten, die sich freiwillig untersuchen lassen wollten.

Auch das erste antivenerische Gericht, das *Gericht über eine Prostituierte*, in dem es um die syphilitische Ansteckung eines Rotarmisten ging, entstammte dem militärmedizinischen Milieu. Den Plot lieferte der Militärarzt Aleksandr Šimanko (geb. 1888), während sein Kollege, Aleksandr I. Akkermann (geb. 1894), die Textvorlage für das Gericht schrieb. Seine Premiere feierte das Gericht 1921 in Moskau im großen Auditorium des Polytechnischen Museums, das schon in der vorrevolutionären Zeit als Veranstaltungsort diverser populärwissenschaftlicher Vorlesungen und Dispute genutzt wurde. Zum Vortragssaal, der über 900 Sitzplätze verfügte [29], strömten die Neugierigen in Massen. Selbst die Vertreter der Parteispitze und Intellektuelle wie David B. Rjazanov (1870–1938) gehörten zum Publikum [30].

**Figure 3:**
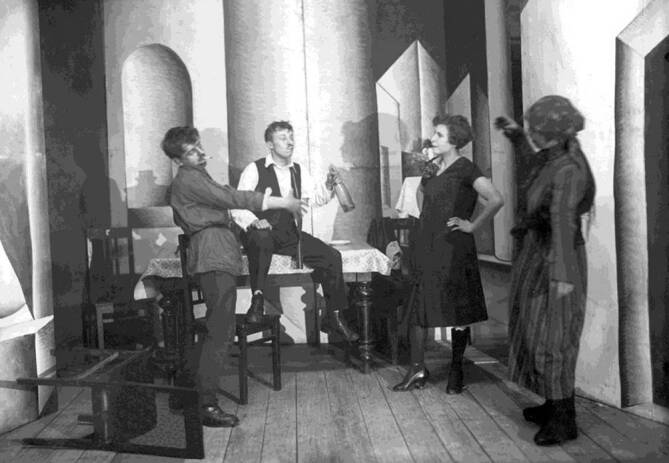

Auf der Anklagebank sitzt in diesem frühen Gerichtsstück eine junge Frau namens Anatasija Zaborova, bei der das Vollbild der hochansteckenden Sekundärsyphilis diagnostiziert wurde (Abb. 3; [31]). Sie wird beschuldigt, den 20-jährigen Rotarmisten Ivan Krest’janov mit Syphilis angesteckt zu haben. Krest’janov und Kazakov, ein Kamerad aus seinem Regiment, sollen von der jungen Frau in einem Stadtpark angesprochen worden sein, während sie auf einer Bank gesessen und eine geraucht hatten. Das Mädchen war so entzückend, dass sie sie nach Einbruch der Dunkelheit nach Hause begleiteten, wo Krest’janov auch über Nacht blieb. Dass er es mit einer Prostituierten zu tun hatte, will das ahnungslose Ansteckungsopfer erst am nächsten Morgen gecheckt haben, als sie von ihm Geld verlangte. Drei Wochen später bemerkte er ein knötchenförmiges Geschwür auf seinem Glied.

Während der Befragung der beiden Rotarmisten leugnet die Angeklagte ihr Verschulden vehement. Die Indizien und Beweise sind jedoch erdrückend. Aus den Vernehmungen von Zeugen geht hervor, dass sie bereits in der vorrevolutionären Zeit Anschaffen ging und den berüchtigten gelben Schein besaß. Soziale Not, erbliche Belastung und schwere Schicksalsschläge sollen sie dazu gedrängt haben, ihren Körper und ihre Seele zu verkaufen. Sie, Tochter eines Trinkers, musste nach dem Tod ihres Vaters bei einer reichen Familie die Anstellung als Dienstmädchen annehmen, woraufhin der Sohn ihres Arbeitgebers sie zum Beischlaf verführt und geschwängert haben soll. Nachdem die Sache aufflog und das Mädchen vor die Tür gesetzt wurde, geriet es auf die schiefe Bahn. Um ihr Neugeborenes vor dem Hungerstod zu bewahren, gab Zaborova das Kind in die Pflege und begann zu prostituieren. Am Ende hat ihr Kind trotzdem nicht überlebt und sie ihr Leben völlig „ruiniert“. Die große Oktoberrevolution gab ihr zwar eine Chance für den Neuanfang, als ihr eine Stelle bei einem staatlichen Betrieb vermittelt wurde. Auf der Arbeit traf sie aber auf Leute, die über ihre Vergangenheit Bescheid wussten und wurde von ihnen solange bedrängt und beleidigt, bis sie den Job quittierte. So kehrte sie zur Prostitution zurück.

Nach der Befragung von Zeugen fängt das hygieneaufklärerische Kernstück der Inszenierung an. Der ärztliche Sachverständige kommt zu Wort und erläutert in Detail die Ansteckungswege, Symptomatik, Phasenverlauf und Therapie der Lues. Dann vergleicht er, was der Kläger, die Angeklagte und die Zeugen über die mutmaßliche Ansteckung ausgesagt haben mit dem, was an Befunden Krest’janovs und Zaborovas tatsächlich vorliegt. Sein Fazit: Krest’janov konnte sich in der Tat nur bei Zaborova angesteckt haben.

Daraufhin folgt in den Reden des Anklägers und Verteidigers die politische Einordnung und ethische Würdigung des Falls. Dabei vollzieht sich eine charakteristische Diskursverschiebung, indem sie nicht mehr in der Syphilis, sondern in der Prostitution eine „soziale Krankheit“ ausmachen und sich an der diffizilen Dialektik des Phänomens abarbeiten, dass diese nach dem Sieg der großen Oktoberrevolution immer noch fortexistiert, obwohl „dass Scherbenhaufen der alten bourgeoisen Welt von der Sturmflut des Arbeiter-und-Bauern-Blutes weggespült worden ist“ [31, S. 40]. Es gilt aus der Sicht des Anklägers, über den Lebenswandel Zaborovas vor und nach der großen Revolution separat zu urteilen [31, S. 40]. Über eine Frau, die unter Bedingungen sozialer Ungleichheit im Zarenreich Anschaffen gehen musste, um Hunger und Armut zu entkommen, dürfe man den Stab nicht brechen. Wer aber nach dem Sieg der großen Revolution, der alle Bürger „vor dem mächtigen Zaren namens Hunger gleichgemacht hat“, der Unzucht nicht abschwört, sei mit aller Strenge des Gesetzes zu bestrafen. Die junge Frau habe sich nicht nur dem kollektiven Aufbau der neuen Gesellschaftsordnung entzogen, sondern sei auch unserer Arbeiter-und-Bauern-Armee in den Rücken gefallen, indem sie sich ahnungslosen Rotarmisten anbot und sie wissentlich durch die syphilitische Ansteckung kampfunfähig machte [31, S. 40].

Der Verteidiger will aber die dichotome Sicht auf die beiden Lebensabschnitte seiner Mandantin nicht teilen. Sei doch das Bordell „ein Gift, welches für immer vergiftet“ [31, S. 51]. Da ihr die alte bourgeoise Welt alle Kräfte geraubt habe, müsse man nicht über sie, sondern über die alte bourgeoise Welt Gericht halten. Der Verteidiger bringt noch ein weiteres Argument in Stellung, um die Fortexistenz sozialer Krankheiten in der Sowjetrepublik zu rechtfertigen: die genetische Disposition der Delinquentin:

Von ihren Eltern erbte Zaborova Willensschwäche, Hysterie, Neigung zur Mutlosigkeit. In ihrem Blut ist Alkohol – ein scharfes Gift, das ihre Brüder vergiftete, ihren Vater ins Grab brachte und sie schwer behinderte [31, S. 53].

Weinend bestätigt Zaborova das Plädoyer ihres Anwalts, wonach das Leben selbst sie dazu zwang, auf den Straßenstrich zu gehen und schwört, nichts von ihrer Krankheit gewusst zu haben. Das Gericht erklärt zwar in seiner Urteilsbegründung Zaborova der verdeckten Prostitution für schuldig, hält aber den Anklagepunkt der vorsätzlichen Ansteckung für unbewiesen. „In Anbetracht ihrer proletarischen Herkunft, der schweren Erschütterungen während der Zeit des zaristischen Regimes, den verantwortungslosen Umgang mit ihr der Menschen in ihrem Umfeld und ihre erbliche Belastung“ beschließt das Gericht, Zaborova zu stationären Syphilisbehandlung einzuweisen und das Volkskommissariat für Sozialfürsorge zu beauftragen, eine Beschäftigungsmöglichkeit für sie zu schaffen, die ihr ein Existenzminimum garantiert.

Das *Gericht über eine Prostituierte* war ein großer Erfolg. Allein in Moskau wurde es im Jahr 1921 150-mal aufgeführt. Doch damit alle Moskauer Arbeiter diesem Gericht beiwohnen konnten, brauchte man 1000 Aufführungen [32]. So hat der Volkskommissar für Gesundheitsschutz, zugleich Vorsitzender des Zentralrats für die Bekämpfung der Prostitution, Nikolaj A. Semaško (1874–1949) angeordnet [33], die Gerichtsinszenierungen speziell am 8. März, dem Internationalen Frauentag, vielerorts aufzuführen [34].

Prostituierte waren selbstverständlich nicht die einzige Ansteckungsquelle der Geschlechtskrankheiten. Wie erwähnt, infizierten sich die meisten Menschen im Bekannten- bzw. Familienkreis. Dieses Problem verhandelt das *Gericht über den Bürger Kiselev* aus der Feder der Frauenärztin Elizaveta B. Demidovič (geb. 1868). Vor Gericht muss sich in diesem Stück aus dem Jahr 1923 der 29-jährige Geschäftsmann Pavel Kiselev verantworten, der sich noch zu Kriegszeiten mit Gonorrhö ansteckte und seine Behandlung immer wieder unterbrach, sobald die akuten Symptome – Juckreiz und eitriger Ausfluss – nachließen. Tripper gilt in seinem Bekanntenkreis als eine Lappalie wie die Masern, die ein jeder echte Mann durchgemacht haben muss [25, S. 20]. 1918 nach seiner Demobilisierung heiratet er Maria, reizende Tochter einer Gymnasiallehrerin. Das Mädchen ist von Geburt an dazu erzogen worden, keusch und rein zu sein. Aus ihren großen blauen Augen strahlen Unschuld, Frische und Gesundheit. Doch schon wenige Monate nach der Hochzeit machen sich verdächtige blaue Schatten unter ihren schönen Augen bemerkbar. Maria bringt zwar noch einen gesunden Jungen zur Welt. Etwa 4 Tage nach der Geburt entwickelt er aber die Symptome einer Gonokokkenkonjunktivitis und erblindet, weil die hinterwäldlerische Hebamme die Krankheit nicht erkennt und die damalige Standardbehandlung mit Silbernitrat-Augentropfen ausbleibt. Der herbeigerufene Arzt klärt die Eltern über die Ursache der eitrigen Bindehautentzündung auf. Maria, die von der Trippererkrankung ihres Mannes nichts wusste, erfährt zudem, dass sie keine Kinder mehr bekommen kann. Das Verhältnis des Ehepaars ist irreparabel beschädigt. Vor lauter Stress wird Maria stillunfähig und ihr Kind verstirbt an einer Magen-Darm-Erkrankung. Daraufhin erhängt sich Maria in ihrem Zimmer an einem Gardinenseil.

Marias Mutter zerrt nun ihren Schwiegersohn vor Gericht und verlangt eine harte Strafe. Wäre aber eine solche bei einer Tripperansteckung verhältnismäßig? Die zentrale Spannungslinie dieses Gerichtsstücks bildet offenbar die Diskrepanz zwischen dem Ruf der „kleinen Schwester der Syphilis“ Gonorrhö als einer harmlosen Unpässlichkeit und der realen Gefahr ihrer schweren Verläufe und Komplikationen. Gegen die Bagatellisierung von Tripper anzukämpfen ist offenbar das volkspädagogische Hauptanliegen des Gerichtsstücks. Diesem Zweck dient v. a. die Rede des Experten, der den Verlauf des unbehandelten Trippers bei Mann und Frau in düstersten Farben schildert und vor der lebensbedrohlichen gonokokkenverursachten Endokarditis dringend mahnt.

Unter Eindruck dieser abschreckenden Schilderungen schließt sich der öffentliche Ankläger der Forderung der Mutter an, Kiselev mit 3 Jahren Haft zu bestrafen und stempelt ihn als „körperlich und seelisch verseucht“ ab [25, S. 47]. Seine Rede kulminiert in einer zornigen Ansprache an das Publikum des Gerichtsspiels, in der der Schätzung des ärztlichen Experten zufolge 85 % aller Männer an Tripper erkrankt sind oder waren:

Denkt daran: Wer nicht erwischt ist, ist fürs Gericht kein Dieb. Doch im tiefsten Inneren euren Gewissens müsst ihr euch selbst gestehen, dass ihr hier alle Verbrecher seid: Verbrecher gegen sich selbst, gegen eure Familien, gegen unseren Staat. Kommt zur Besinnung und handelt, bevor es zu spät ist [25, S. 47]!

Der Verteidiger plädiert aber für den Freispruch und bittet das Gericht, seinen Mandanten als Opfer unzureichender Sexual- und Hygieneaufklärung zu begnadigen. Schuld sei seine Mutter, die ihn als kleinen Jungen mit dem Märchen vom Klapperstorch hinters Licht geführt habe, statt ihn in die wahren Umstände von Zeugung einzuweihen. Schuld seien die Schullehrer, die sich beim Thema venerische Krankheiten in einen dicken Mantel des Schweigens gehüllt haben. Schuld sei das Nachtleben der Großstadt „mit ihren zynisch entblößten Frauen, ihren Café-chantants und Operettchen, die den Sexualtrieb des noch unreifen Jugendlichen vorzeitig geweckt haben“ [25, S. 50]. Am allerschlimmsten sei aber die gesellschaftliche Bagatellisierung der Gonorrhö gewesen. Der Artikel 155 stelle bewusste Übertragung einer schweren venerischen Krankheit unter Strafe. War aber die Gonorrhö in Kiselevs Augen eine schwere Krankheit? Keineswegs! Wusste er doch über die Gonorrhö lauter „dumme Märchen“ [25, S. 55]. Eine bewusste Übertragung einer schweren venerischen Krankheit dürfe unter diesen Umständen nicht inkriminiert werden. Das Gericht schwenkt schließlich auf die Linie der Verteidigung ein. In Anbetracht seiner Wissensdefizite wird gegen Kiselev lediglich eine öffentliche Rüge verhängt und Einweisung zur Zwangsbehandlung angeordnet. Die Hintertür, die der Anwalt Kiselevs bei seiner geschickten Verteidigungsstrategie im fiktiven Strafverfahren erfolgreich benutzte, wurde aber für reale Gerichtsverhandlungen im gleichen Jahr verschlossen. Auf Initiative des Volkskommissariats für Gesundheitswesen verschwanden aus dem Satz „bewusste Übertragung einer schweren venerischen Krankheit“ im Artikel 155 die Wörter „bewusste“ und „schwere“ [35, 36].

## Die antivenerischen Theaterstücke

Die ersten sowjetrussischen Aufführungen von antivenerischen Dramen sind aus dem Jahr 1920 dokumentiert. Am Anfang griff man auf vorrevolutionäre Erfahrungen zurück. Eugène Brieuxs Bühnenstück *Les Avariés* wurde im Moskauer dramatischen Theater Fedor A. Koršs (1852–1923) bereits im November 1905 aufgeführt [37]. Der gleiche Plot kam 1913 mit dem von Jakov A. Protazanov (1881–1945) gedrehten Film *Das Brandzeichen vergangener Vergnügungen (ärztliche Schweigepflicht;* Klejmo prošedšich naslaždenij/Vračebnaja tajna) auch auf die Leinwand [38]. So lag es nahe, nach der Revolution an diese Erfolge von *Les Avariés* direkt anzuknüpfen. Schon im März 1920 wurde das Drama in der Stadt Wologda gespielt. Eine Lokalzeitung berichtete, dass das Publikum, größtenteils Arbeiter und Rotarmisten, sie begeistert aufnahm: Solche Stücke seien heute aktueller denn je und jedenfalls nützlicher als der *Eugen Onegin* Aleksandr S. Puškins [39]. Im Dezember 1920 fand im Staatlichen Theater von Smolensk die Uraufführung der russischen Adaptation des Stücks *Mit Schande bedeckt *(Zaklejmennye pozorom) statt, die vom Sanitärarzt Michail D. Utënkov (1893–1953) verfasst wurde [40]. Mitte der 1920er-Jahre kamen dann auch die ersten Stücke russischer Autoren auf die Bühne, die den französischen Prätext in vielfacher Weise variierten.

Das Grundgerüst der Figurenkonstellation und die Triebfeder des Plots eines typischen antivenerischen Bühnenstücks bildet die Infektkette einer Geschlechtskrankheit. Der Protagonist lässt sich in einem verhängnisvollen Augenblick der Schwäche und Sinnesrausches oder nach einem alkoholischen Exzess mit einer Dirne ein, stürzt sich in eine Affäre mit einer hübschen Kollegin oder kann den verlockenden Reizen seiner Nachbarin nicht widerstehen. Er holt sich dabei eine Geschlechtskrankheit, meistens Syphilis, woraus schwere Auswirkungen auf seine Ehe und Nachkommenschaft resultieren. Der Schock sitzt tief und fordert den Protagonisten heraus, die eigenen Wertvorstellungen und Überzeugungen auf den Prüfstand zu stellen. Warum um alles in der Welt hat das Unglück ausgerechnet ihn getroffen? Üblicherweise ist der tragische Held kein notorischer Draufgänger, der ausschweifendes Geschlechtsleben führt, sondern zumindest in der Außenwahrnehmung ein durchaus rechtschaffener Mann. Eine solche Figurenkonzeption findet man bereits bei Brieux prototypisch vor, dessen Hauptheld, der wohlhabende Notar Georges Dupont, darüber fassungslos ist, dass das Unglück ausgerechnet ihn traf, obwohl er doch allen Freuden versagte und sich eine einzige Entgleisung an seinem Junggesellenabend erlaubte. Brieux ging es offenbar darum, Geschlechtskrankheiten als ein gesamtgesellschaftliches Problem zu situieren, das jeden betrifft und nicht bloß ein Stigma bestimmter sozialer Randgruppen ist. Utënkov folgt hier Brieux bei seiner Adaptation von *Les Avariés* und präsentiert einen wohlhabenden russischen Kaufmann. Allerdings wird er auf der Bühne als verwöhntes Muttersöhnchen vorgeführt, um seine klassenfeindliche bourgeoise Natur hervorzuheben [41].

In originär sowjetischen Theaterstücken ist die Hauptfigur in der Tendenz wieder eher positiv markiert, häufig ist der Syphilitiker ein integres Komsomol-Mitglied, gefeierter Bestarbeiter oder Gewerkschaftsführer: ehrlich, zuverlässig, linientreu. Geplagt von Zukunftsängsten und Gewissensbissen, Schamgefühlen und Selbstmordgedanken ist der Protagonist eines antivenerischen Theaterstücks hin- und hergerissen: Er fürchtet gemieden und geächtet zu werden, sollte er seine geheime Krankheit öffentlich machen. Sein Familienleben und sein Ansehen im Betrieb stehen auf dem Spiel. Während der Protagonist mit seinem inneren Konflikt sich auf diese Weise als der eigene Gegenspieler präsentiert und mit sich kämpft, bleibt der eigentliche Antiheld und Thementräger des Dramas, die bleiche Spirochäte, naturgemäß unsichtbar und doch in Gestalt ihrer Handlanger – Halbweltdamen, Prostituierten und Zuhältern – personifiziert. Eine weitere Verbündete der Krankheit ist häufig die Kurpfuscherin, die mit ihren obskuren Praktiken verhindert, dass der Kranke dem richtigen Behandlungsregime unterworfen wird. Als zweite Hauptfigur fungiert in diesen Dramen die unwissentlich angesteckte Ehefrau des Protagonisten, die typischerweise ein Kind erwartet oder seine unschuldige Braut, die von der Erkrankung ihres zukünftigen Ehepartners nicht erfahren darf. Die dramaturgische Funktion des Mentors und Helfers erfüllt in der Regel ein Arzt. Er ist eine Porte-parole-Figur und moralische Instanz zugleich, die den Protagonisten über die heimtückische Natur seiner Krankheit schonungslos aufklärt, eindringlich an sein Gewissen appelliert und beschwört, zur Verhütung des neuen Unglücks nicht ungeheilt in die Ehe einzutreten oder auf den Geschlechtsverkehr mit seiner Ehefrau zu verzichten. Die Konfiguration wird schließlich durch 2–3 Randfiguren abgerundet, die häufig stark typisierte Witzcharaktere sind und die Geschichte aufhellen. Dieses einfache Schema wird aber von Stück zu Stück verschiedentlich variiert und erweitert.

Eine eigenartige Wendung nimmt das Grundschema im Versdrama von A. I. Šapiro und O. K. Kudra *Vier Kreuze* (Četyre kresta; 1923), das als Karikatur auf das bürgerliche Melodram konzipiert ist [42]. Der syphilitische Protagonist Ivan Lepeško bekleidet hier sogar den respektablen Posten eines Leiters des Bezirkslebensmittelkomitees. Als ehemaliger Zarenoffizier gehört er allerdings in die Kategorie der sog. „Altspezialisten“ (Specy), die im Arbeiter-und-Bauern-Staat dringend benötigt sind, aber als klassenfremdes Element stets mit Argwohn und Misstrauen beäugt werden. Sein gesundes Äußeres ist ebenso trügerisch wie seine Loyalität gegenüber dem bolschewistischen Regime. Lepeško verschweigt seiner Braut Sonja seine Erkrankung und feiert die Hochzeit mit ihr. Wenig später erleidet Sonja eine Fehlgeburt. Ihr Gesundheitszustand verschlechtert sich zusehends. Wie sich am Ende herausstellt, steckt sich sogar ihre Mutter durch die Benutzung des gemeinsamen Geschirrs an. Eines Tages rät Sonjas behandelnder Arzt, der bösen Verdacht schöpft, zu einem Wassermann-Test. Auf dem Befundzettel, den sie aus dem Labor mitbringt, prangen 4 Kreuze. Der herbeigerufene Arzt klärt die Familie über die furchtbare Bedeutung dieser Zeichen auf und schildert wortreich alle drei Verlaufsphasen der Syphilis und den körperlichen Verfäll während ihrer letzten Phase: „Die Fäulnis nimmt die letzte Kraft/Man lebend wird dahingerafft/Oft plagt den Kranken die Parese/Oft ist die Nase ganz verwesen“ [42, S. 22].^3^ Der Arzt greift sämtliche Topoi auf, die die zeitgenössische Syphilidophobie befeuern und schließt mit den befürchteten Symptomen der Gehirnerweichung: „So modert langsam er,/Verliert seine Organe,/Verfällt dem Wahnsinn/Ohne es zu ahnen“ [42, S. 22].^4^ Dieses Aufklärungsgespräch hinterlässt bei Sonja einen solch tiefen Eindruck, dass sie wild ausrastet und ihren Ehemann als gemeinen Lügner und Mörder verflucht. Als sie wieder zu Besinnung kommt und ihn zurückrufen lässt, ist es zu spät. Der antiquierte Ehrenkodex des Ex-Zarenoffiziers gebietet Lepeško nach seinem Revolver zu greifen und sich zu erschießen, womit er abermals beweist, wie schlecht er in die neue Gesellschaftsordnung hineinpasst.

**Figure 4:**
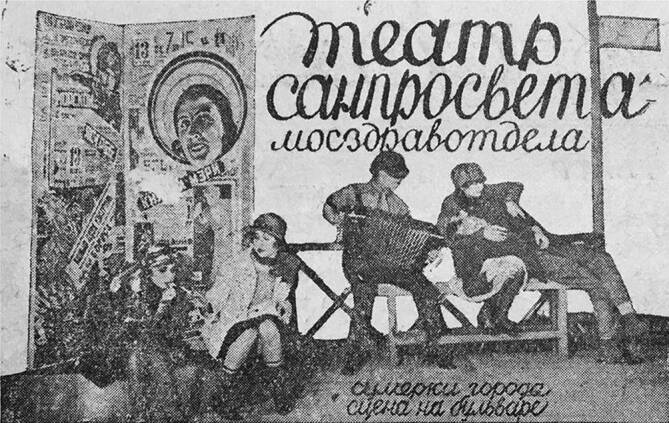

Die wohl originellste dramaturgische Interpretation des antivenerischen Grundschemas bietet *Das Dämmerlicht der Stadt *(Sumerki goroda) von Aleksandr Ventcel’ und Grigorij Goler (Abb. 4 und 5; [43]). Die aus 6 Episoden bestehende Fallgeschichte zeigt die dunklen und hellen Seiten der Großstadt, führt den Zuschauer durch die Lasterhöhlen und Lotterbetten des Moskauer Rotlichtmilieus in die Lichtstätten der sanitären Aufklärung und Reinigung, Arbeiterclubs und venerologische Dispensaires. Fedor ist ein einfacher Bauer, der das schöne Dorfmädchen Lisa über alles liebt und vom Familienglück mit ihr auf seinem Bauernhof träumt. Die beiden haben sich bereits als Jugendliche die ewige Liebe geschworen. Der ausgebrochene Bürgerkrieg durchkreuzte aber ihre Hochzeitpläne. Während Fedor in Budënnys Kavalleriebrigade gegen die Weißgardisten tapfer kämpfte, zog Lisa nach dem Tod ihrer Eltern nach Moskau zu ihrer großen Schwester Sof’ja, die dort seit Jahren lebt und ihr einen Job an der Fabrik „Befreite Arbeit“ vermittelt hat. Jetzt, nachdem die Kämpfe zu Ende sind, kehrt Fedor von der Front zurück und die Sehnsucht nach Lisa führt ihn in die größte russische Metropole.

**Figure 5:**
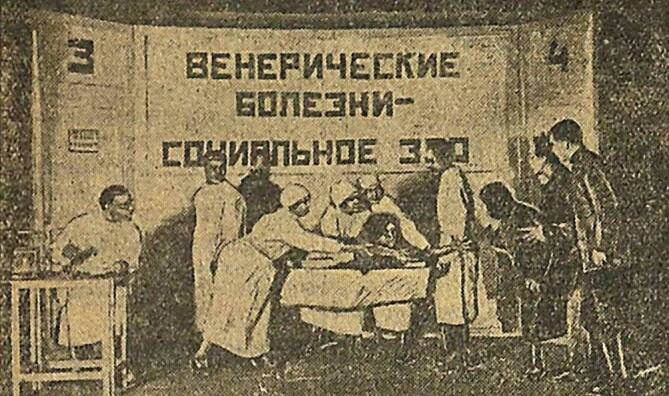

Zeitgleich schmieden in einem Moskauer Amüsierbetrieb drei dubiose Geschäftsmacher Šmarenkov, Svetkov und Saška-Amerikaner den Plan, ein illegales Etablissement zu eröffnen. Auch Sof’ja, Lisas große Schwester, ist mit von der Partie. Ihre Stelle bei der „Befreiten Arbeit“ hat sie längst hingeschmissen und ist im kriminellen Sumpf des Rotlichtmilieus tief versunken. Das Gespräch kommt auf die bildhübsche Lisa. Šmarenkov will auch sie für sein neues Bordell verführen und überredet Sof’ja, ihre Schwester zu verkaufen. Am nächsten Tag gelingt es Sof’ja, die ahnungslose Lisa in eine düstere Spelunke zu locken, wo sich das Zuhältertrio schon die Hände reibt. Anfangs lässt sich Lisa auf ihre plumpe Anmache nicht ein. Plötzlich klopft es aber an der Tür. Fedor, Lisas geliebter Bräutigam, ist ihr auf die Spur gekommen und bittet um Einlass. In dieser schmutzigen Gesellschaft von ihm gesehen zu werden, wäre eine Schande. Lisa ist bereit, jeden Preis zu zahlen, damit ihr diese Szene erspart bleibt und lässt sich von Šmarenkov durch die Hintertür entführen. Während dessen wird Fedor von Cvetkov und Saška-Amerikaner abgefüllt. Sie geben ihm den Rat, seine Braut aus dem Kopf zu schlagen. Denn sie sei bloß eine gewöhnliche Dirne. Der untröstliche Fedor begießt seinen Gram mit Wodka. Da setzt sich Sof’ja zu ihm hin, um mit ihm „auf Liebe“ anzustoßen. Schließlich geht er mit ihr aufs Zimmer, ohne in ihr Lisas Schwester erkannt zu haben. Kurz darauf werden die Gäste der Spelunke von einer Razzia der Miliz überrascht.

In der 5. Episode sitzt dann die ganze Zuhälterbande auf der Anklagebank des Volksgerichts und wird zu mehrjährigen Haftstrafen verurteilt. Sof’ja bekommt für die wissentliche Ansteckung Fedors 1 Jahr und 2 Monate Gefängnis. Das Gericht erkennt jedoch an, dass sie Opfer schwerer sozialer Not sei und setzt die Strafe auf Bewährung aus. Das Drama endet im Warteraum des venerologischen Dispensaires, wo Lisa und Fedor zufällig aufeinanderstoßen und sich in die Arme fallen: „Lisa du!?“ „Fedor!“ Es stellt sich heraus, dass sie beide wegen der gleichen Schanddiagnose hierhergekommen sind, und Fedor sagt resigniert: „Lass uns also zusammen verfaulen.“ In diesem Augenblick lässt sie ein jämmerliches Geschrei zusammenzucken. Auf einer Trage wird durch die Klinikflur eine verwirrte Frau geschleppt, die närrisch kichert und ruft: „Komm Bübchen, komm doch her, ich singe dir ein Lied! Lisa! Lisa! Verkauft habe ich sie, verkauft!“ Lisa erkennt mit Entsetzen ihre große Schwester und Fedor seine Ansteckungsquelle. Fedor: „Schwester? Deine Schwester? […] Von ihr habe ich das … Was für ein Ende. So wird man auch uns einmal hinaustragen.“ Doch Lisa widerspricht ihm leidenschaftlich. Die Syphilis sei nämlich ein heilbares Übel, wenn man es richtig behandelt. Das hätten ihr die Ärzte gesagt. Man könne wieder von vorne anfangen und alles werde irgendwann wie früher sein, verspricht sie. „Nein“, antwortet Fedor fest entschlossen, „bloß nicht wie früher. Wir beide machen alles auf neue Art!“

## Rezeption und Wirkung

Die Rezeption der sanitären Gerichte durch die Zuschauer ist besser als die der sanitären Theaterstücke dokumentiert. Sie Avancierten zu einer Massenattraktion der 1920er-Jahre. So waren bei einer Inszenierung des *Gerichts über eine Prostituierte* im Jahr 1923 bis zu 2000 Arbeiter und Angestellte der Waggonwerkstatt der Kursk-Eisenbahn anwesend [44]. Ihre Popularität verdankten die Agitationsgerichte nicht zuletzt ihrem melodramatischen Charakter [10, S. 52]. Gerichtsinszenierungen riefen heftige Anteilnahme beim Publikum hervor, welches das Spektakel mit spontanem Beifall, Aufschreien und Zwischenrufen begleitete und oftmals in Tränen ausbrach [45]. Das Interesse ließ selbst bei mehrstündigen Aufführungen nicht nach. Auf dem Kurbad am Eltonsee schaute das Publikum einem Gerichtsspiel sogar an zwei Terminen insgesamt 9 h lang wie gebannt zu [46].

Die Realitäts- und Bodenhaftung des Gerichtstheaters als Genre wurde dadurch fundiert, dass die Verfasser der Gerichtsstücke sich zuvor von realen Gerichtsprozessen inspirieren und diese nachspielen ließen. Hierbei wurde versucht, die Grenze zwischen realer Rechtspraxis und Fiktion zu verwischen, so dass den Zuschauern häufig nicht bewusst war, dass sie einer theatralen Inszenierung beiwohnten. Theatral bezeichnet man ein Setting, wo „etwas oder jemand bewusst exponiert oder angeschaut wird“ [47]. Im Arrangement eines inszenierten Gerichtsstücks verschwand jedoch die spezifische Theatralität des Theaters hinter dem theaterhaften Vollzug eines gerichtlichen Rituals. Organisatorisch und schauspieltechnisch musste man allerdings einige elementaren Dinge beachten. Während die Aufführungen als echte Gerichtsverhandlungen angekündigt wurden, waren die Schauspieler angehalten, so authentisch und glaubwürdig wie möglich aufzutreten, als spielten sie sich selbst. Nachdem jedoch der Vorhang fiel und die zu Haftstrafen verurteilten Delinquenten die Bühne frei verlassen durften, kam es immer wieder zu enttäuschten Reaktionen aus dem Publikum [48]. Um eine nachhaltige performative Wirkung der Aufführungen nach ihrem Ende sicher zu stellen, entwickelten die Praktiker der antivenerischen Gerichte diverse Strategien. So wurden die Agitgerichte am Moskauer Venerologischen Dispensaire als auswärtige Sitzungen des Volksgerichts getarnt und in Abwesenheit des Angeklagten abgehalten [48]. Das Publikum erfuhr, dass die Identität des Delinquenten, der im Zuschauerraum unerkannt sitze, aufgrund der ärztlichen Schweigepflicht geheim bleiben müsse, um ihn vor psychischer Traumatisierung zu bewahren. Somit konnten die Zuschauer nicht ahnen, dass sie an einer theatralen Gerichtsaufführung teilnahmen. Häufig spielten die Patienten venerologischer Dispensaires sich selbst, ohne zu wissen, dass sie vor Schauspielrichtern und -Anklägern auf der Bühne stehen. Endete das Gerichtsstück mit einem milden Urteil, beispielsweise der „öffentlichen Rüge“, dann flog die Fiktion überhaupt nicht auf, so dass die performative Kraft des Schuldspruchs über die Dauer der Aufführung hinaus anhielt [49].

Übrigens war der Unterschied zwischen Realität und Fiktion unter damaligen Verhältnissen geringer als man sich vielleicht heute denken würde. Auch in frühsowjetischen Realgerichten agierten als Richter und Anwälte meistens keine professionellen Juristen, sondern Arbeiter und Bauern ohne spezielle Ausbildung [50, 51]. Als Pflichtverteidiger wurden oft die Arbeitskollegen der Angeklagten, insbesondere Kultur- oder Gewerkschaftsfunktionäre des Betriebs, beigeordnet. So konnte ein und derselbe Gewerkschaftssekretär einer Fabrik heute der Pflichtverteidiger sein und morgen den Pflichtverteidiger spielen. Die gespielte und reale Gerichtsbarkeit konnte selbst im Hinblick auf die juristische Wirksamkeit des Richterspruchs zusammenfallen, wenn z. B. eine fiktiv ausgesprochene öffentliche Rüge eine durchaus reale performative Wirkung entfaltete. Dieses Einswerden zwischen Sein und Schein bereitete allerdings auch eigene Probleme: Häufig fiel es unter diesen Bedingungen den Veranstaltern schwer, Kandidaten für die Rollen der Prostituierten und Syphilitiker zu rekrutieren [32, S. 6].

Doch es ging nicht nur darum, die Grenze zwischen Realität und Fiktion zu verflüssigen. Im postrevolutionären schöpferischen Theater war man bestrebt, auch die Trennung zwischen passiv bleibenden Zuschauern und aktiv Handelnden auf der Bühne, die sog. „vierte Wand“, aufzuheben [6, S. 15]. Die Idee einer durch eine imaginäre Wand vom Zuschauerraum getrennten, autark abgeschlossenen eigenen Realität des Schauspiels auf der Bühne wurde im realistischen Theater des späten 19. Jahrhunderts zu seinem zentralen schauspieltechnischen Prinzip erhoben [52]. In theaterreformerischen Kreisen der Avantgarde setzte sich aber das gegenläufige Bestreben durch, die „vierte Wand“ einzureißen, da in ihr ein Abbild der politischen Verhältnisse der bürgerlichen Klassengesellschaft gesehen wurde [5, S. 145]. Ein fiktiver Gerichtsprozess war für dieses Konzept wie geschaffen. Das fing schon damit an, dass die Zuschauer sich von ihren Sitzen zu erheben hatten, wenn die Mitglieder des Gerichts den Saal betraten. Am Ende der Verhandlung durften sie häufig über das Strafmaß abstimmen [49, 53]. Die maximale volkserzieherische Wirkung versprach man sich, wenn das Gericht Milde zeigte und eine öffentliche Rüge verhängte. Das setzte wiederum gestiegene Anforderungen an den öffentlichen Verteidiger, der für einen Meinungsumschwung im Publikum zugunsten des Angeklagten kämpfen musste, indem er einer individuellen Schuldzuschreibung und Bestrafung die Forderung entgegensetzte, die Gesellschaft als Ganzes zu verurteilen. Häufig gelang es in der Tat, die Zuschauer von ihrer Kollektivschuld zu überzeugen und zu einem milderen Urteil zu bewegen, als es die Anklage forderte [44].

Gegen Ende der 1920er-Jahre ließ die Popularität des Genres des Agitgerichts langsam nach. Sein volkserzieherischer Charakter wirkte übertrieben plakativ und für manchen künstlerisch versierten Theoretiker der sanitären Aufklärung sogar „primitiv“ [54]. Die Leitung des Moskauer Theaters der Sanitären Kultur bemühte sich deswegen um die Erweiterung seines Repertoires. Ein dramaturgisch hochwertiges Theaterstück bot größere gestalterische Spielräume, um seine agitatorisch-propagandistischen Ziele zu verschleiern und dadurch größeren „Erziehungseffekt“ zu erzielen [55]. Ließ sich das zu vermittelnde hygienische Wissen nicht direkt in die Handlung einflechten, bestand immer noch die Möglichkeit, einen extradiegetischen Erzähler einzuführen. In der Regel war es der Arzt, der beispielsweise als Conférencier zu Beginn der Aufführung oder in den Pausen auftrat, um das Bühnengeschehen in das rechte medizinische Licht zu rücken.

**Figure 6:**
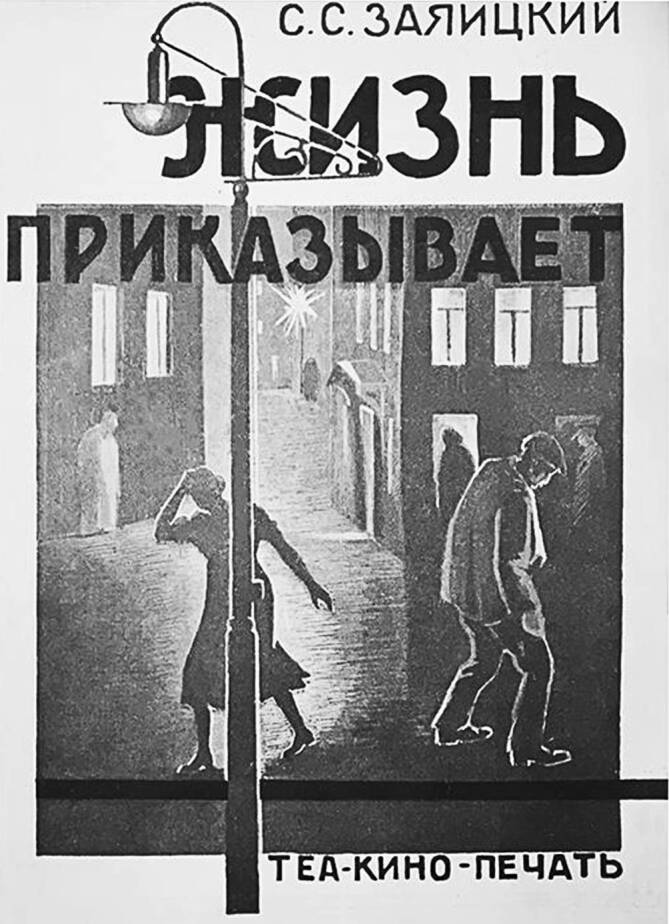

Um festzustellen, ob diese Botschaften beim Publikum tatsächlich ankamen, wurden die Aufführungen regelmäßig evaluiert. Erhalten sind beispielsweise die ausgewerteten Fragebogen zum Stück Sergej S. Zajaickijs (1893–1930) *Das Leben befiehlt*, die zeigen, dass die Zuschauer die Hauptidee des Stücks erfassen konnten (Abb. 6): Die Syphilis ist heilbar, wenn sie rechtzeitig erkannt und angemessen behandelt wird. Über die Krankheit muss ohne „falsche Scham“ gesprochen werden [56]. Laut Zeitungsberichten waren auch die Aufführungen von *Das Dämmerlicht der Stadt *und* Das Leben befiehlt *ein großer Erfolg [57–60]. Lina S. Nejman (1887–1971), eine Literatur- und Theaterkritikerin, die 1928 *Das Dämmerlicht der Stadt* in einem Arbeiterclub besuchte, hat einige Stimmungen im Zuschauerraum eingefangen. Als das Dorfmädchen Lisa in die Hände der Zuhälterbande gerät, flüstern die Arbeiterinnen aufgeregt: „Jetzt füllen sie sie ab, Halunken!“, „Nun ist sie geliefert!“, „Jetzt schleppt er sie ab – das Mädchen ist verloren“ [61]! Die große Mehrheit des Publikums sind Frauen, viele mit Kind im Schoß oder im Arm. Manche müssen in den Gängen stehen, weil der Saal rammelvoll ist. Man hört Gelächter, Schluchzen und Zwischenrufe und hat das Gefühl, die Bühne und das Publikum seien eins. In der Pause wird über die Figuren des Stücks aufgeregt geredet und gestritten, als seien es reale Personen. Einige Frauen beginnen über die Seitensprünge von Kollegen und Kolleginnen untereinander zu tuscheln: „Habt ihr gehört, auch bei uns in der Fabrik …“ Nach der Vorstellung verlässt das Publikum den Arbeiterclub in tief andächtiger Stille. „Was lief da gerade?“ – fragt ein neugieriger Passant. „Ein venerisches Schauspiel“, antwortet eine Zuschauerin ernst und fügt hinzu: „Ein lehrreiches Stück“ [61].

Die Öffnung zum Zuschauerraum wurde auch bei einigen antivenerischen Dramen angestrebt. Am Theater der sanitären Aufklärung von Lugansk wurden sogar Sitzungen des Künstlerrats des Theaters Teil der Inszenierung. Nach der Aufführung durften die Zuschauer mitentscheiden, ob ein Stück ins ständige Repertoire aufgenommen werden soll [63]. Meistens blieb man jedoch Traditionen und Sehgewohnheiten des realistischen Sozialdramas treu und ließ die „vierte Wand“ undurchlässig, so dass eine direkte Publikumspartizipation kaum eine Rolle spielte.

Die genaue Zahl der aufgeführten Theaterstücke zum Thema Geschlechtskrankheiten auf den Bühnen der frühen Sowjetunion lässt sich nicht ermitteln, aber allein das Moskauer Theater der Sanitären Kultur spielte im Jahr 1927 210 Stücke mit einer Gesamtzahl von 160.000 Zuschauern in Moskau und Umgebung [57], wobei die Hälfte des Repertoires auf die Schauspiele über die venerischen Krankheiten entfiel [64].

Allerdings teilte die Theaterkritik nicht immer die Begeisterung der Zuschauer oder die Meinung der Protagonisten der Hygieneaufklärung, dass Geschlechtskrankheiten für die sowjetische Dramaturgie einen geeigneten Stoff und eine zündende Inspirationsquelle boten [65]. Während man die Schaffung einer proletarischen Kultur und Befreiung der Künste von bürgerlichen Stereotypen proklamierte und forderte, die herkömmlichen ästhetischen Konventionen in Frage zu stellen, blieben viele Kulturschaffenden dem bildungsbürgerlichen Kunstverständnis doch verhaftet. Wer mit „Fidelio“ und „Schwanensee“ ästhetisch alphabetisiert wurde, konnte mit dem Tanz der kleinen Spirochäten wenig anfangen. So schrieb der bekannte russische Theaterkritiker Viktor Ėrmans (1888–1858) über die Premiere einer Adaption von Victor Margueritte (1866–1942) *Prostituée *enttäuscht: „Im Ergebnis – ein großer, wertvoller Roman von sozialer Bedeutung verwandelte sich in e i n s a n i t ä r e s S t ü c k“ [66]. Aufgrund dieser Imageprobleme waren nur wenige professionelle Schriftsteller bereit, sich mit hygienischen und damit tabuisierten „schmutzigen“ Themen zu befassen. Sanitäre Theaterstücke wurden immer wieder als „naturalistisch“ kritisiert. So monierte die Theaterkritikerin Kasatkina, dass im Stück *Das Dämmerlicht der Stadt *die Szene im venerologischen Dispensaire so realitätsgetreu inszeniert wurde, dass sie abstoßend wirkte. Auch die Schilderung der Amoralität und Obszönität des Moskauer Rotlichtmilieus verfehle ihr Ziel, weil sie eher verführt als brandmarkt [67]. Während gerade solche Szenen das Massenpublikum begeistert mitfiebern ließ, missfielen sie den Protagonisten kultureller Revolution. Der futuristische Dichter Semen I. Kirsanov (1906–1972) beispielsweise äußerte abschätzig: „Sanprosvet ist gezwungen, Pfuscharbeiten mit Liebesintrigen aufzuführen, weil es die Agitation scheut“ [68].

## Schluss

Die Hygienepropaganda in der frühen Sowjetunion nutzte verschiedene Formate: Vorträge und Dispute, Plakate und Ausstellungen, Bücher und Broschüren. Keine dieser herkömmlichen Formen der Volksaufklärung konnte aber das Publikum in der Weise begeistern und mitreißen wie die theatrale Dramatisierung hygienischen Wissens auf der Bühne. Erst recht galt dies für tabubehaftete anrüchige Realitäten wie Geschlechtskrankheiten und Prostitution, die während der 1920er-Jahre zum zentralen Topos der sanitären Aufklärung avancierten.

Das Agitationsgericht v. a. erwies sich auf diesem Feld als „pädagogisch wirksames Lehrtheater“. Nicht zuletzt verdankte es seinen Erfolg dem auf Einheit von Zeit, Ort und Handlung geschlossenen Handlungsaufbau, der die Fokussierung auf einen zentralen Konflikt ermöglichte. Das Genre des Agitationsgerichts war in einer Oszillation von Realität und Fiktion begründet. Einerseits speiste sich ihre Faszinationskraft aus der „unhintergehbar theatralen Dimension des Rechtsprechens“ selbst [69]. Anderseits zeichnete sich das Gerichtstheater durch Strategien der „Enttheatralisierung“^5^ bzw. „unmarkierten Theatralität“ aus [5, S. 156]. Inszenierte und juristisch wirksame Gerichtsprozesse, Gerichtstheater und Justizschauspiel waren für das Publikum oft ununterscheidbar. Das Schauspiel wurde nicht mehr als eine aus der normalen Realität ausgegliederte und zeitlich abgegrenzte zweite Realität wahrgenommen, weil die Fiktion in der genuinen Theatralität des Gerichts aufging. Damit wurden Gelingensbedingungen dafür geschaffen, dass der gespielte Richterspruch reale performative Wirkung entfalten konnte.

Unter künstlerischen Gesichtspunkten waren die Gerichtsspiele dramatischen Theaterstücken allerdings unterlegen. Die Zahl der Letzteren nahm deswegen ab Mitte der 1920er-Jahre zu. Beim Thema Geschlechtskrankheiten und Prostitution war es naheliegend, literarisch und schauspieltechnisch an die Tradition des realistischen Sozialdramas anzuknüpfen, wie sie Russland Aleksandr N. Ostrovskij (1823–1886) oder Maksim Gor’kij (1868–1936) repräsentierten. Bühnenstücke, die dabei entstanden sind, ähnelten gleichzeitig in ihren Deutungsmustern westeuropäischen Vorbildern theatraler Syphilisaufklärung. Die Figur der Prostituierten wurde pathologisiert, so dass Krankheitsbild und -träger nahezu zusammenfielen [71]. In avantgardistischen Künstlerkreisen waren diese Aufführungen jedoch wegen ihres melodramatischen oder naturalistischen Charakters starker Kritik ausgesetzt. Doch genau diese Qualitäten der Aufführungen waren die Grundlage ihrer sozialen Funktion und Wirkung: Es ging darum, die Menschen zu berühren und die gesellschaftlichen Schamschwellen herabzusetzen, damit über die Geschlechtskrankheiten „ohne falsche Scham“ gesprochen werden konnte. Denn eben im scheinheiligen kollektiven Schweigen über die „geheimen Krankheiten“ wurde das zentrale Hindernis ihrer Bekämpfung ausgemacht.

Die Zahl der Inszenierungen von antivenerischen Gerichten und Dramen nimmt Anfang der 1930er-Jahre ab. Zum einen ist das mit dem Wandel der Politik im Kampf gegen die Prostitution zu erklären. Wenn sich die bolschewistische Regierung während der NÖP bemühte, die Prostituierten mittels verschiedener sozialer Programme in die sowjetische Gesellschaft zu integrieren, setzten sich Ende der 1920er-Jahre die repressiven Maßnahmen wieder durch [72]. Zum anderen wurde 1934 in der Sowjetunion der „sozialistische Realismus“ zur verbindlichen Methode der Literatur und Kunst erklärt, was zu einem Paradigmenwechsel innerhalb der Hygieneaufklärung führte. Im sozialistischen Realismus, der sich über eine Ästhetik des Idealtypischen und Wesentlichen definierte, durfte es keine antagonistischen Konflikte mehr geben, weil diese für die sozialistische Wirklichkeit aufgrund der Beseitigung antagonistischer Klassengegensätze „untypisch“ geworden sind. Geschlechtskrankheiten und Prostituierte hatten in einer solchen „Wirklichkeit“ selbstverständlich nichts mehr zu suchen und ihre Darstellung konnte während des Großen Terrors in den späten 1930er-Jahren als politische Provokation und Verunglimpfung der sowjetischen Wirklichkeit schwere Konsequenzen nach sich ziehen. 1934 wurde am Moskauer Institut für Sanitäre Aufklärung unter dem Titel *Sanitäre Aufklärung und der Zweifrontenkampf* eine Broschüre verfasst, die einen Rundumschlag gegen eine ganze Reihe führender Autoritäten auf diesem Gebiet darstellte, die als mechanizistische oder menschewistisch-idealistische Abweichler abgestempelt wurden. Unter anderem wurde der Arztschriftsteller Boris S. Sigal (1893–1983) für seine Aussage scharf gerügt, dass Syphilis auf dem Lande durch demobilisierte Soldaten verbreitet werde:

Verleumdung! Zunächst einmal gibt es bei uns keine „Soldaten“ mehr. Es gibt nur noch Rotarmisten. Zweitens wird dem Autor wohl bekannt sein, dass unsere Rote Armee, im Gegensatz zu allen Armeen kapitalistischer Länder, keineswegs eine Brutstätte venerischer Krankheiten und der Syphilis ist. Ganz im Gegenteil ist unser Rotarmist eine kulturelle Kraft auf dem Dorf [73].

Die sanitären Theaterstücke hatten nicht das Kranke oder Ungesunde, sondern die Gesundheit und den nahenden Sieg über die Krankheiten in Szene zu setzen. Zu bekämpfen waren nunmehr die „eingebildeten Krankheiten“, Hypochondrien, die bei der Erfüllung und Übererfüllung der 5‑Jahres-Pläne der Kollektivierung und Industrialisierung des Landes im Wege zu stehen schienen. Mit Beginn des Zweiten Weltkriegs wurde das Thema des Kampfes gegen die Geschlechtskrankheiten angesichts der „beispielslosen Heldentums“ des sowjetischen Volkes an der Front und im Hinterland gar tabuisiert [74].

## Footnotes

1 Ausführlich zum Wandel der Sexualmoral am St. Petersburger Beispiel vgl. Lebina [20].

2 Ausführlich zur Legalisierung des Schwangerschaftsabbruchs im Kontext der frühsowjetischen Frauenpolitik vgl. Goldman [21]; Speziell zu rechtlichen Aspekten vgl. Rjasencev [22]; Vgl. auch Solov’ev [23].

3 Übersetzt aus dem Russischen von Igor J. Polianski.

4 Übersetzt aus dem Russischen von Igor J. Polianski.

5 Zum Konzept der Enttheatralisierung des Theaters siehe: Fischer-Lichte [70].

## References

[1] Linse U, Schuller A, Heim N (1987). Über den Prozeß der Syphilisation, Körper und Sexualität um 1900 aus ärztlicher Sicht. Vermessene Sexualität.

[2] Lasowski W (1982). Syphilis. Essai sur la littérature française du XIXe siècle.

[3] Schonlau A (2005). Syphilis in der Literatur – Über Ästhetik, Moral, Genie und Medizin (1880–2000).

[4] Ellenbrand P (1999). Die Volksbewegung und Volksaufklärung gegen Geschlechtskrankheiten in Kaiserreich und Weimarer Republik.

[5] Frölicher G, Caduff M, Heine S, Steiner M (2015). Aktive Partipation oder inszenierte Mitsprache? Sowjetische Agitationsgerichte der 1920er Jahre. Die Kunst der Rezeption.

[6] Frölicher G, Sasse S (2015). Gerichtstheater. Drei sowjetische Agitgerichte.

[7] Frölicher G, Sasse S, Krämer S, Schmidt S (2016). Das ‚richtige‘ Sehen: Zeugen im sowjetischen Gerichtstheater. Zeugen in der Kunst.

[8] Sasse S (2009). Wortsünden. Beichten und Gestehen in der russischen Literatur.

[9] Sasse S (2003). Gerichtsspiele. Fiktive Schuld und reale Strafe im Theater und vor Gericht. Kunst als Strafe. Zur Ästhetik der Disziplinierung.

[10] Cassiday JA (2000). The enemy on trial. Early Soviet courts on Stage and screen.

[11] Wood EA (2005). Performing justice: agitation trials in early Soviet Russia.

[12] Narodeckij AZ (1928) Sanitarnoe prosveščenie čerez scenu. Tezicy doklada na XI Vsesojuznom s’ezde bakteriologov, ėpidemiologov i sanitarnych vračej. RGANTD 178/5/4, Bl. 4

[13] Einleitung im Findbuch Nr. 5 des Bestandes 178. RGANTD

[14] Brief K. Stanislavskijs an die Künstlerisch-Dramatische Theaterstudio des Volkskommissariats für Gesundheitswesen, 25. Juli 1921. RGANTD 178/5/111, Bl. 2–8

[15] Kedrov G (1928). Teatr Sanprosveta Mosgorzdravotdela. Sovremennyj Teatr.

[16] Stenogramm der Konferenz der Theater des Volkskommissariats für Gesundheitswesen in der Ukraine und Moskau, 5. Dez. 1932. RGANTD 178/5/8. Bl. 4

[17] Vetrov V, Petrov V (1926). Agitsud i živaja gazeta v derevne.

[18] Polianski IJ (2016). Das Schweigen der Ärzte. Eine Kulturgeschichte der sowjetischen Medizin und ihrer Ethik.

[19] Bernstein FL (2007). The dictatorship of sex: Lifestyle advice for the Soviet masses.

[20] Lebina NB, Lebina NB, Škarovskij MV (1994). V otsutstvie oficial’noj prostitucii. Prostitucija v Peterburge (40-e gg. XIX v.–40-e gg. XX v.).

[21] Goldman WZ (1993). Woman, the State and Revolution. Soviet Family Policy and Social Life 1917–1936.

[22] Rjasencev VA (1971). Semejnoe pravo.

[23] Popov AA, Judin BG (1998). Aborty v Rossii. Bioėtika: principy, pravila, problemy.

[24] Lebina NB (1999). Povsednevnaja žizn’ sovetskogo goroda: normy i anomalii. 1920–1930 gody.

[25] Demidovič EB (1923). Sud nad gr. Kiselevym, po obvineniju ego v zaraženii ženy ego gonoreej, posledstviem čego bylo ee samoubijstvo.

[26] Williams C (2018). Health and Welfare in St. Petersburg 1900–1941.

[27] Müller-Dietz H (1956). Die Bekämpfung der Geschlechtskrankheiten in der Sovjetunion und in der Sovjetischen Besatzungszone Deutschlands.

[28] Kubanova AA, Martynov AA, Vlasova AV (2017). Vekovoj opyt otečestvennoj dermatovenerologiii. Ėtapy razvitija kožno-venerologičeskoj pomošči naseleniju. Vestinik Dermatol Venerol.

[29] Semenova OV (2015). Politechničeskij muzej. Nauka Pervych Ruk.

[30] III sessija VCIK. Prenija po proektu ugolovnogo kodeksa (1922). Izvestija VCIK 108:2

[31] Akkermann AI (1922). Sud nad prostitutkoj. Delo gr. Zaborovoj po obvineniju ee v zanjatii prostituciej i zaraženii sifilisom kr-ca Krest’janova. Gosudarstvennoe izdatel’stvo.

[32] Ėdel’štejn A, Akkermann AI Sud nad prostitutkoj (1922). Neskol’ko slov o sanprosvetsudach. Delo gr. Zaborovoj po obvineniju ee v zanjatii prostituciej i zaraženii sifilisom kr-ca Krest’janova.

[33] Panin S (2005). „Prodažnaja ljubov“ v Sovetkoj Rossii (1920e gody). Vestnik Evrazii. Istorija. Istoričeskie Nauki.

[34] Bor’ba s prostituciej (1923). Izvestija VCIK 148:4

[35] Ob izmenenijach i dopolnenijach Ugolovnogo kodeksa RSFSR (1923) Sobranie uzakonenij i raporjaženij Rabočego i krest’janskogo pravitel’stva 48:881

[36] Prigradov-Kudrin K (1923). Dobavlenija i popravki k ugolovnomu kodeksu. Izvestija VCIK.

[37] Osipova OK (2013). O russkich perevodah p’esy Ėžena Brijë „Porčennye“. Drevnjaja I Novaja Romanija.

[38] Klejmo prošedšich naslaždenij (1913). Sine-Fono 5:48

[39] Volžskaja Kommuna, 10.03.1920, http://vkonline.ru/toprint/82991.html. Zugeriffen: 01.12.2019

[40] Utenkov MD (1924). Zaklejmennye pozorom. P’esa.

[41] Rabkor Š-O (1924). Zaklejmennyj pozor [Rezension. Rabočij Zritel.

[42] Šapiro AI, Kudra OK (1923). Četyre kresta. Sanitarnaja p’esa.

[43] Ventcel’ A, Goler G (1926). Sumerki goroda. P’esa.

[44] Opal’nyj (1923). Obšestvennyj sud. Gudok.

[45] Narodeckij AZ (1930) Pjat’ let raboty teatra Moszdravotdela. RGANTD 178/5/7, Bl. 12

[46] Vedernikov PM (1924). San-sudy v postanovke na osnove kollektivizma, kak put’ k aktivirovaniju samodejatel’nosti mass (po sledstvennym materialam).

[47] Warstat M (2005). Theatrale Gemeinschaften. Zur Festkultur der Arbeiterbewegung 1918–33.

[48] Ioffe LS. Obščestvennyj sud nad bol’nym tripperom, zarazivšim ženu i rebenka. RGANTD 178/5/127, Bl. 21

[49] Ašavskij M (1924). Sud nad sifilitikom. Pravda.

[50] Vinničenko OJ, Filonova OI (2013). Modernizacija sudebnoj sistemy v period nėpa. Izd-vo Kurganskogo gos.

[51] Sineokij OV (2008). Advokatura kak institut pravovoj pomošči i zaščity.

[52] Mangan M (2013). The drama, theatre and performance companion.

[53] Narodeckij AZ (1928) Sanitarnoe prosveščenie čerez scenu. Tezicy doklada na XI Vsesojuznom s’ezde bakteriologov, ėpidemiologov i sanitarnych vračej. RGANTD 178/5/4, Bl. 7

[54] Berman FJ (1928) Podgotovka sanprosvetrepertuara. RGANTD 178/5/5, Bl. 7

[55] Narodeckij AZ (1928) Sanitarnoe prosveščenie čerez scenu. Tezicy doklada na XI Vsesojuznom s’ezde bakteriologov, ėpidemiologov i sanitarnych vračej. RGANTD 178/5/4, Bl. 3

[56] Fragebogen zur Untersuchung der Wahrnehmung der Zuschauer des Stücks von S.S. Zajaickijs Das Leben befiehlt. RGANTD 178/5/6, Bl. 1–7

[57] Kedrov G (1927). Central’nyj teatr Sanprosveta. Žizn’ Iskusstva.

[58] Kedrov G (1928). „Žizn’ prikazyvaet“. Sovremennyj Teatr.

[59] Teterin (1930). Takoj teatr nužen rabočim. Gudok.

[60] Moskovskij teatr na linii (1927) Gudok 222:3

[61] Nejman L (1928). Po rabočim klubam. Izvestija VCIK.

[62] Zajaickij SS, 1929, Žizn’ prikazyvaet. Drama. Tea-kino-pečat’, Moskva

[63] Istomin V (1930). Luhans’kyj teatr sanosvity. Sil’s’kyj Teatr.

[64] Narodeckij AZ (1930) Pjat’ let raboty teatra Moszdravotdela. RGANTD 178/5/7, Bl. 8

[65] Volkonskaja SN, Bermal FJu, Kanevsij LO (1934) Sdelat’ sanitarnuju kul’turu dostojaniem millionov. RGANTD 178/1/59, Bl. 7

[66] Ėrmans V (1925). Prostitutka. Zerkal’nyj teatr Ėrmitaža. Novyj Zritel’.

[67] Kasatkina A (1927). Sumerki goroda. Pravda.

[68] Semen Kirsanov, Äußerung in Wiedergabe Vladimir Majakovskijs. In: Majakovskij V (1961) Polnoe sobranie sočinenij v trinadcati tomach. T. 13, Pis’ma i drugie materialy. Goslitizdat Moskva, S. 126

[69] Vismann C (2011). Medien der Rechtsprechung.

[70] Fischer-Lichte E, Willems H (2009). Enttheatralisierung des Theaters als Theatralisierung des öffentlichen Lebens. Soziologische Theorie und Zeitdiagnose.

[71] Lazardzig J (2002). Inszenierung wissenschaftlicher Tatsachen in der Syphilisaufklärung. „Die Schiffbrüchigen“ im Deutschen Theater zu Berlin (1913). Hausarzt.

[72] Lebina N (2015). Sovetskaja povsednevnost’: normy i anomalii. Ot voennogo kommunizma k bol’šomu stilju.

[73] F. J. Berman, red. Sanitarnoe prosveščenie v bor’be na dva fronta. 1934. RGNTD 178.1.60, S. 37.

[74] Sitzungsprotokolle der Repertoirekommission des Theaters der Sanitären Kultur, 19. Juni 1945. RGANTD 178/1/278, Bl. 4–8RS

